# REV-ERBα Participates in Circadian SREBP Signaling and Bile Acid Homeostasis

**DOI:** 10.1371/journal.pbio.1000181

**Published:** 2009-09-01

**Authors:** Gwendal Le Martelot, Thierry Claudel, David Gatfield, Olivier Schaad, Benoît Kornmann, Giuseppe Lo Sasso, Antonio Moschetta, Ueli Schibler

**Affiliations:** 1 Department of Molecular Biology and NCCR Frontiers in Genetics, Sciences III, University of Geneva, Geneva, Switzerland; 2 Laboratory of Experimental and Molecular Hepatology, Department of Internal Medicine, Medical University of Graz, Graz, Austria; 3 Laboratory of Lipid Metabolism and Cancer, Consorzio Mario Negri Sud, Santa Maria Imbaro (Chieti) and Clinica Medica Murri, University of Bari, Italy; University of Cambridge, United Kingdom

## Abstract

In mammals, many aspects of behavior and physiology, and in particular cellular metabolism, are coordinated by the circadian timing system. Molecular clocks are thought to rely on negative feedback loops in clock gene expression that engender oscillations in the accumulation of transcriptional regulatory proteins, such as the orphan receptor REV-ERBα. Circadian transcription factors then drive daily rhythms in the expression of clock-controlled output genes, for example genes encoding enzymes and regulators of cellular metabolism. To gain insight into clock output functions of REV-ERBα, we carried out genome-wide transcriptome profiling experiments with liver RNA from wild-type mice, *Rev-erb*α knock-out mice, or REV-ERBα overexpressing mice. On the basis of these genetic loss- and gain-of-function experiments, we concluded that REV-ERBα participates in the circadian modulation of sterol regulatory element-binding protein (SREBP) activity, and thereby in the daily expression of SREBP target genes involved in cholesterol and lipid metabolism. This control is exerted via the cyclic transcription of *Insig2*, encoding a trans-membrane protein that sequesters SREBP proteins to the endoplasmic reticulum membranes and thereby interferes with the proteolytic activation of SREBPs in Golgi membranes. REV-ERBα also participates in the cyclic expression of cholesterol-7α-hydroxylase (CYP7A1), the rate-limiting enzyme in converting cholesterol to bile acids. Our findings suggest that this control acts via the stimulation of LXR nuclear receptors by cyclically produced oxysterols. In conclusion, our study suggests that rhythmic cholesterol and bile acid metabolism is not just driven by alternating feeding–fasting cycles, but also by REV-ERBα, a component of the circadian clockwork circuitry.

## Abstract 2

The nuclear receptor REV-ERBα shapes the daily activity profile of Sterol Response Element Binding Protein (SREBP) and thereby participates in the circadian control of cholesterol and bile acid synthesis in the liver.

## Author Summary

The mammalian circadian timing system has a hierarchical architecture: a central pacemaker in the brain's suprachiasmatic nucleus (SCN) synchronizes subsidiary oscillators present in most peripheral cell types. In both SCN neurons and peripheral cells, circadian oscillators are thought to rely on two negative feedback loops. A major feedback loop involves the two cryptochromes CRY1 and CRY2 and the two period proteins PER1 and PER2, which serve as transcriptional repressors for their own genes. An accessory feedback loop couples the expression and activity of the transcriptional activators CLOCK and BMAL1 to the expression of cryptochrome and period proteins. The orphan nuclear receptor REV-ERBα is a key player in this accessory feedback loop, in that it periodically represses *Bmal1* transcription. In liver, molecular clocks mediate the temporal gating of metabolic processes. Here we demonstrate that hepatocyte clocks participate in the control of cholesterol and bile acid homeostasis. According to this scenario, REV-ERBα shapes the circadian expression pattern of insulin-induced gene 2 (INSIG2), a resident protein of the endoplasmic reticulum that interferes with the proteolytic activation of sterol response element binding proteins (SREBPs). In turn SREBPs govern the rhythmic expression of enzymes with key functions in sterol and fatty acid synthesis. The circadian production of sterols (in particular oxysterols) may engender the cyclic activation of LXR nuclear receptors, which serve as critical activators of *Cyp7a1* transcription. CYP7A1, also known as cholesterol 7α-hydroxylase, catalyzes the rate-limiting step in bile acid synthesis.

## Introduction

Virtually all light-sensitive organisms from cyanobacteria to humans possess circadian clocks that allow them to anticipate environmental changes and thus to adapt their behavior and physiology accordingly. The mammalian circadian timing system has a hierarchical structure, in that daily light-dark cycles phase-entrain the master clock in the suprachiasmatic nucleus (SCN), which in turn synchronizes subsidiary oscillators in most peripheral cells [1],[2]. How the SCN signals to peripheral organs to entrain their oscillators is still poorly understood, but daily feeding–fasting cycles, body temperature oscillations, and SCN-controlled circadian hormone rhythms appear to play a central role in this process [3]–[6].

In both the SCN and in the periphery, the circadian oscillator is thought to be based on a negative transcriptional/translational feedback loop involving multiple clock components, notably members of the Period (PER1, PER2) and Cryptochrome (CRY1, CRY2) protein families. These proteins rhythmically inhibit their own transcription by interfering with the transactivation potential of CLOCK/NPAS2∶BMAL1 heterodimers [2],[7]. The circadian transcription of the gene encoding the nuclear orphan receptor REV-ERBα is regulated by a similar mechanism, in that it is activated by CLOCK∶BMAL1 and repressed by PER∶CRY complexes. The periodic accumulation of REV-ERBα provokes the cyclic repression of the essential clock gene *Bmal1* [8]. REV-ERBα thus couples the so-called positive and negative limbs of the molecular oscillator. Transcriptome profiling studies on wild-type and mice defective for core oscillator components have uncovered that the core clock also regulates the rhythmic activity of circadian output genes, which account for overt circadian rhythms in physiology and behaviour [9]–[13]. For example, CLOCK∶BMAL1 in the liver drives the cyclic expression of the transcription factors of the proline- and acid-rich basic region leucin zipper (PAR bZIP) family albumin D-site-binding protein (DBP), thyrotroph embryonic factor (TEF), and hepatic leukemia factor (HLF), which in turn regulate the transcription of genes encoding detoxification enzymes [14].

Hepatic cholesterol and bile acid synthesis have long been known to be subject to circadian regulation [15]–[17]. During the activity phase, when animals absorb food, large amounts of cholesterol are converted into bile acids, which are secreted into the gut as emulsifiers of lipids. To ensure cholesterol homeostasis, the intracellular cholesterol pool has to be replenished. Conversion of cholesterol into bile acid must therefore be tightly coordinated during the day, and transcription of genes encoding key enzymes of cholesterol metabolism are indeed highly circadian.

Cholesterol availability is sensed in membranes of the endoplasmic reticulum (ER) by the sterol regulatory element-binding protein (SREBP)-cleavage activating protein (SCAP)-INSIG-SREBP complex. SREBP members belong to the basic helix-loop-helix leucine zipper (bHLH-LZ) transcription factor family. While SREBP-2 controls genes involved in cholesterol synthesis, such as *Hmg-CoA reductase* (*Hmgcr*) [18], SREBP-1c mainly regulates genes implicated in lipogenesis, such as the gene encoding fatty acid synthase (FAS) [19]. SREBPs are synthesized as inactive precursors anchored in the ER membrane via two transmembrane domains, where they bind to the eight-transmembrane domain protein SCAP [20]. The ER-resident proteins INSIG-1 [21] and INSIG-2 [22] are responsible for the sterol-dependent retention of the SCAP-SREBP complex in the ER. SCAP and INSIG-2 are cholesterol and oxysterol sensors, respectively, and can both initiate formation of the SCAP-SREBP-INSIG ternary complex depending on sterol availability [23]. At low sterol concentrations, the SCAP-SREBP complex is released from INSIG proteins and transported to Golgi vesicles, where the N-terminal SREBP moiety is cleaved. The processed SREBP protein then translocates to the nucleus and activates the transcription of genes encoding enzymes involved in fatty acid and cholesterol synthesis [24].

Cytochrome P450 7α-hydroxylase (CYP7A1) is the rate-limiting enzyme in bile acid synthesis. *Cyp7a1* transcription is governed by many transcription factors, notably by several nuclear receptors [25],[26]. For example, the two nuclear receptors Liver-X-Receptor (LXR) and Farnesoid-X-Receptor (FXR) regulate *Cyp7a1* expression depending on the availability of their ligands, which are themselves derivatives of cholesterol metabolism. LXR binds oxysterols (whose levels parallel those of cholesterol) and activates *Cyp7a1* transcription [27],[28], whereas FXR binds bile acids and represses *Cyp7a1* transcription [29]–[31]. In addition, *Cyp7a1* expression has long been known to oscillate during the day, but the transcription factors implicated in this circadian transcription remained controversial.

Here we show that the orphan nuclear receptor REV-ERBα is a key player in the circadian regulation of cholesterol and bile acid synthesis by influencing rhythmic SREBP activity and *Cyp7a1* expression. Recently, Duez et al. [32] have reported that REV-ERBα-deficient mice have a low bile acid phenotype. Although our study is in keeping with this observation, on the basis of our genetic loss-of-function and gain-of-function studies we propose different molecular pathways accounting for the bile acid phenotype.

## Results

### Identification of Putative REV-ERBα Target Genes by Affymetrix Microarray Analysis

**Figure 1:**
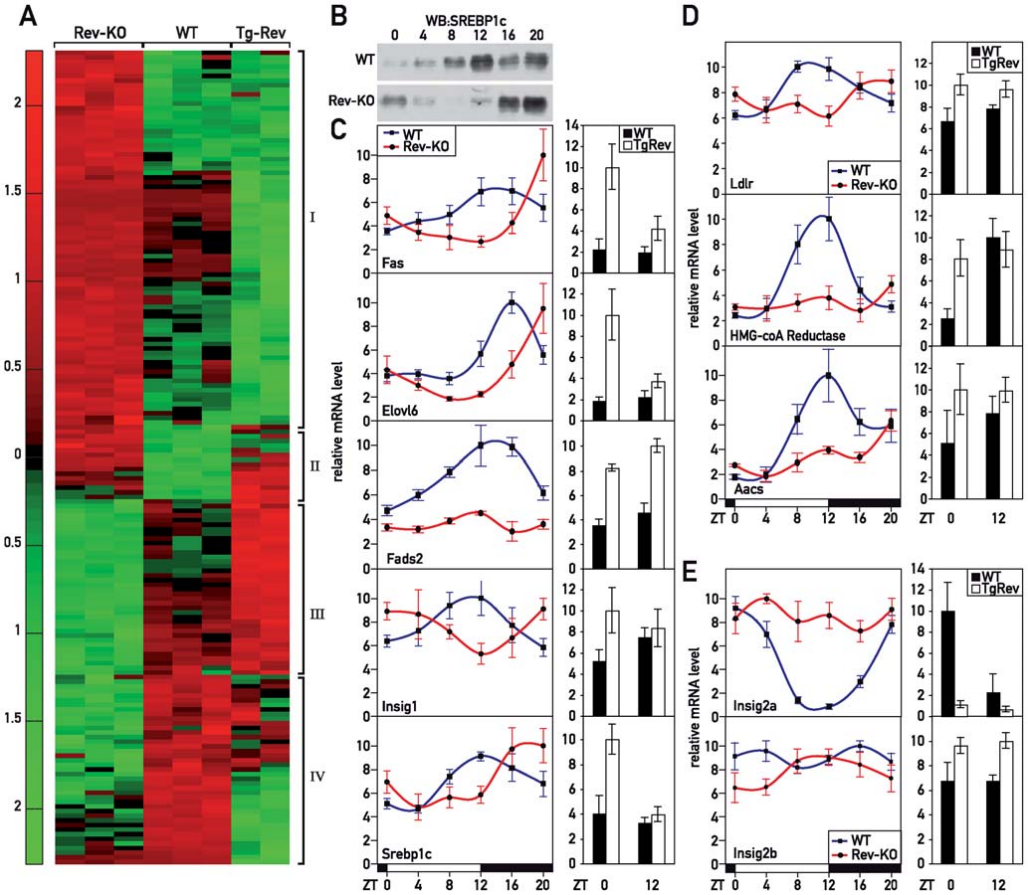
REV-ERBα controls the temporal nuclear accumulation of SREBP and the transcription of SREBP target genes. (A) Affymetrix-microarray analysis of liver RNA from mice of various genotypes. The heat map displays transcripts with differential accumulation in WT, Rev-KO, and TgRev mice. Differentially expressed transcripts of mice with different genotypes are clustered into four classes: class I: Rev-KO>WT≥TgRev; class II: Rev-KO>WT<TgRev; class III: Rev-KO<WT≤TgRev; class IV: Rev-KO<WT>TgRev (see also Figure S2). (B) Temporal accumulation of SREBP1c in liver nuclear extract. (C) Temporal expression of transcripts from selected SREBP1c genes. (D) Temporal expression of transcripts from selected SREBP2 target genes. (E) Temporal expression of *Insig2* transcripts. The data displayed in (C–E) were obtained by quantitative (Q) RT-PCR experiments on whole-cell liver RNA from WT, Rev-KO, and TgRev animals. The data represent the mean±SEM (*n* = 4–6).

To gain insight into the physiological functions of REV-ERBα, we identified REV-ERBα target genes in a genome-wide manner, using Affymetrix microarray hybridization analysis of liver RNA from *Rev-erb*α knock-out (Rev-KO) and wild-type (WT) animals humanely killed at ZT12 (ZT, Zeitgeber time; ZT0, lights on; ZT12: lights off). At this time point the differences in the expression of direct REV-ERBα target genes can be expected to be maximal in Rev-KO versus control animals (see [8] and Figure S1). Because REV-ERBα acts as a transcriptional repressor, transcripts specified by direct target genes should be overrepresented in the Rev-KO animals. Transgenic mice that overexpress hepatic REV-ERBα throughout the day [11] were also examined. In these animals, referred to as TgRev, direct REV-ERBα target genes should be expressed at low levels irrespective of the time of day. Accordingly, indirect target genes with reduced expression in Rev-KO mice should display constant high levels in TgRev animals. The differential expression of genes in the livers of WT, Rev-KO, and TgRev mice is shown in the genome-wide transcriptome profiling experiments displayed in Figure 1A (see lists in Table S1 and S2). As expected, most genes found to be up- and down-regulated in Rev-KO animals were down- and up-regulated, respectively, in TgRev mice (Figure 1A, bracket I and III). Only a subset of genes did not follow this simple scenario and showed equal regulation in both Rev-KO and TgRev animals (Figure 1A, bracket II and IV). Mechanistic models potentially accounting for such less intuitive accumulation profiles are depicted in Figure S2.

REV-ERBα oscillates with a sharp expression peak in WT animals. We thus expected that most of the 76 genes (represented by 97 probe sets) that we identified as up-regulated in Rev-KO animals at ZT12 (Table S1) would show circadian mRNA accumulation profiles in WT animals. For the 60 genes (represented by 81 probe sets) down-regulated in Rev-KO (Table S2) this is not necessarily expected as they probably represent indirect REV-ERBα target genes. So far REV-ERBα has only been described as a transcriptional repressor. Obviously, it cannot be excluded that REV-ERBα might also function as an activator on certain target genes, for example by recruiting co-activators. Indeed, recruitment of both co-activators and co-repressors has been reported for the related nuclear receptor Retinoic acid receptor-related Orphan Receptor α (RORα) [33]. We thus extracted the circadian accumulation profiles of the identified genes from a comprehensive study of genome-wide circadian hepatic gene expression at a 1-h resolution that was recently performed by the Hogenesch laboratory [10] (see also Gene Expression Omnibus GSE11923 [http://www.ncbi.nlm.nih.gov/geo] and http://bioinf.itmat.upenn.edu/circa). Using stringent algorithms, around 1% of the liver transcriptome was circadian in this analysis, whereas 54% (41 transcripts) of the up-regulated and 43% (26 transcripts) of the down-regulated fractions identified in our microarray analysis corresponded to circadian genes (Figure S3). This result confirms that REV-ERBα is predominantly a regulator of circadian gene expression. The circadian phases of most of the up-regulated transcripts corresponded to that expected for direct REV-ERBα targets. The phases of the down-regulated transcripts were less uniform, possibly reflecting several indirect mechanisms.

### SREBP Pathways Are Impaired in *Rev-erb*α Null Mice

Analysis of the microarray data showed that *Rev-erb*α disruption led to the misregulation of several genes involved in lipid metabolism. In particular, known target genes of the cholesterol-sensing transcription factor SREBP [34] were expressed at lower levels in Rev-KO animals (Table S2). This suggested that the INSIG-SCAP-SREBP pathway was temporally misregulated. To examine this conjecture, we assessed the nuclear accumulation of SREBP1c in liver at 4-h intervals around the clock. In WT animals, nuclear SREBP1c protein was highly abundant at ZT12, but barely detectable at ZT0 and ZT4 (Figure 1B). In Rev-KO animals, however, the accumulation of SREBP1c in the nucleus was shifted by 8 h (Figure 1B). This phase-shifted nuclear SREBP1c protein accumulation in Rev-KO mice correlated with the phase-shifted expression of SREBP1c target genes, such as *Fas*, *Elovl6*, *Fads2*, *Insig1*, or *Srebp1c* itself (Figure 1C, left panel). In TgRev mice, these genes were up-regulated at ZT0 and virtually unchanged at ZT12, with the exception of *Fads2* which was up-regulated at both time points (Figure 1C). These findings substantiated the hypothesis that REV-ERBα controlled the temporal expression of SREBP1c target genes. The altered expression of *Hmgcr*, *Low density lipoprotein receptor (Ldlr)*, *and Acetoacetyl-CoA synthetase (Aacs)* in Rev-KO and TgRev animals suggested that the activity of SREBP2 was affected in these animals as well (Figure 1D), in spite of the apparently normal *Srebp2* mRNA accumulation (Figure S4A).

The altered nuclear accumulation of SREBP1c in livers of Rev-KO animals could have been due to a misregulation of its mRNA (and consequently protein) expression (Figure 1C) and/or a delay in the processing and transport of SREBP1c to the nucleus. To investigate the latter possibility, we analyzed whether in Rev-KO mice there were any peculiarities to the SREBP processing machinery that would explain the delay in nuclear SREBP1c accumulation. Whereas *Insig2a* mRNA (coding for the major INSIG isoform in liver) showed a 7-fold circadian amplitude in WT livers, it was indeed expressed at constantly high and low levels in Rev-KO and TgRev mice, respectively (Figure 1E). This suggested that *Insig2a* was a bona fide REV-ERBα target gene. We were unfortunately unable to examine whether corresponding changes also exist for INSIG2a on the protein level, since the two commercial anti-INSIG2a antibodies we purchased did not yield satisfactory results in immunoblot experiments. However, since INSIG2a is known to be a short-lived protein [35], the oscillation of *Insig2a* mRNA very likely also translated to the rhythmic accumulation of INSIG2a protein in ER membranes. The expression of *Scap* mRNA (Figure S4B) and the non-circadian minor *Insig2b* isoform (Figure 1E) were not affected in Rev-KO mice.

### REV-ERBα Regulates SREBP1c Target Genes Independently of the Feeding Regimen

**Figure 2:**
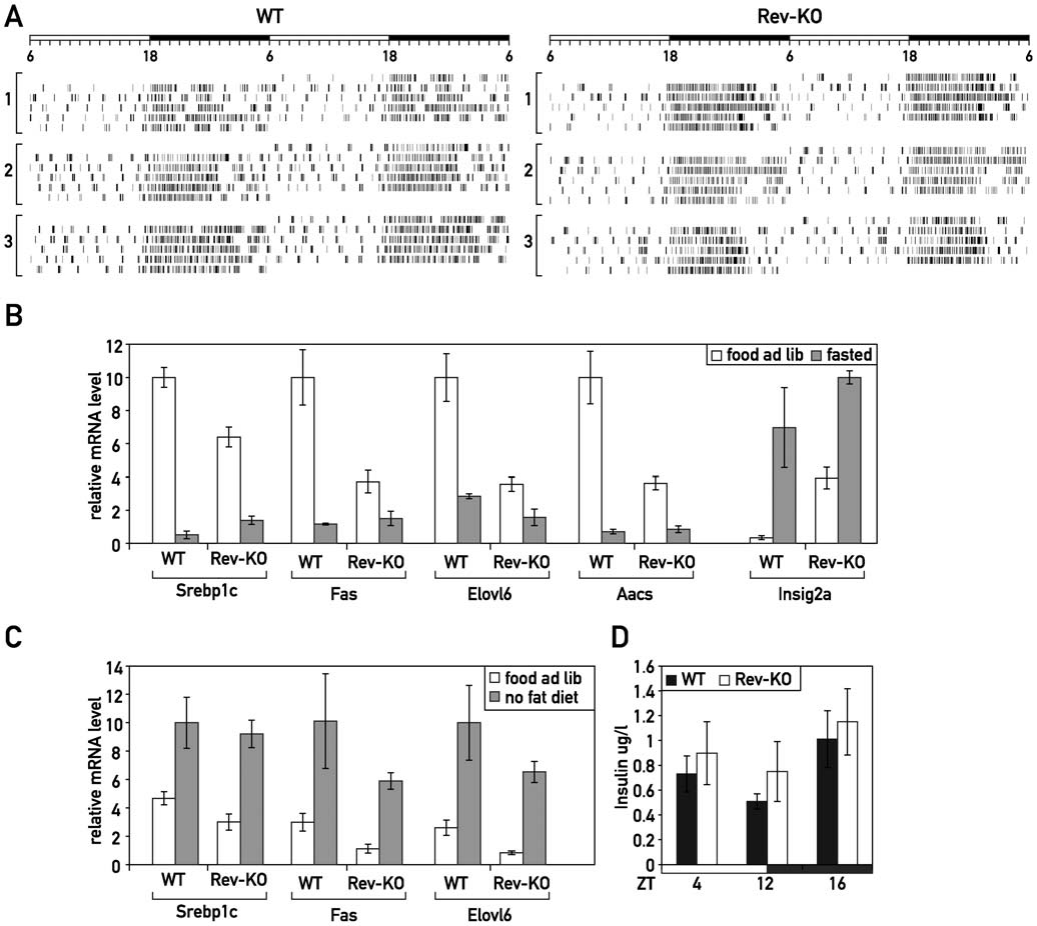
Fasting–feeding regulation of SREBP targeted genes in Rev-KO mice. (A) Feeding activity of three Rev-KO and WT mice as measured by an infrared detection system. (B) Mice that were either fasted for 24 h or (C) fed a high carbohydrate–no fat diet for 18 d were humanely killed at ZT12, and the accumulation of selected mRNAs was measured in WT and Rev-KO (*n* = 4 per each data point) using Q-RT PCR. (D) Insulin measurement from blood samples collected at ZT4, ZT12, and ZT16 (*n* = 5 per each data point). (B–D) data represent the mean±SEM.

As shown above, REV-ERBα controlled the nuclear accumulation of SREBPs and, thus, the expression of SREBP targets. However, it has also been shown that these processes are regulated by fasting–feeding [36], fatty acids [37]–[39], and insulin [40]. Therefore, we wished to determine if the temporal misregulation of the SREBP pathway in Rev-KO mice merely reflected altered feeding rhythms and/or differences in plasma insulin. To this end, we recorded the time of food consumption in WT and Rev-KO animals by an infrared detection system; there were no major differences between the genotypes (Figure 2A).

We then fasted WT and Rev-KO mice for 24 h and measured the expression of selected SREBP target genes that we had found to be regulated in Rev-KO mice fed ad libitum at ZT12 (see Figure 1C). As shown in Figure 2B, fasting-induced repression of SREBP target genes such as *Srebp1c*, *Fas*, *Elovl6*, and *Aacs*, also occurred in the Rev-KO background. Also the fasting-induced up-regulation of *Insig2a* [41] reached similar expression levels in Rev-KO and control animals (Figure 2B). The fold-up-regulation was obviously less dramatic in the Rev-KO because basal expression levels in the fed state were already 10-fold higher than in control animals.

We next fed mice with a high carbohydrate–no fat diet that is known to induce the expression of lipogenic SREBP1c target genes such as *Srebp1c*, *Fas*, and *Elovl6*. Again, we measured the effects at ZT12 in Rev-KO and control mice, and found that diet-dependent regulation was independent of the genotype (Figure 2C). In addition, we did not notice any differences in plasma insulin concentrations between the genotypes at ZT12 (when SREBP target genes are differentially regulated in WT and Rev-KO mice), at ZT4 (the beginning of the day phase), or at ZT16 (the beginning of the night phase) (Figure 2D).

These findings suggest that the regulation of the SREBP pathway is governed by at least two independent processes. In addition to its regulation by fasting and feeding that has been described before, the circadian clock, through rhythmic REV-ERBα accumulation, regulates the SREBP pathway independently of the feeding regimen.

### REV-ERBα Controls Temporal Lipid Accumulation in Blood and Liver

**Figure 3:**
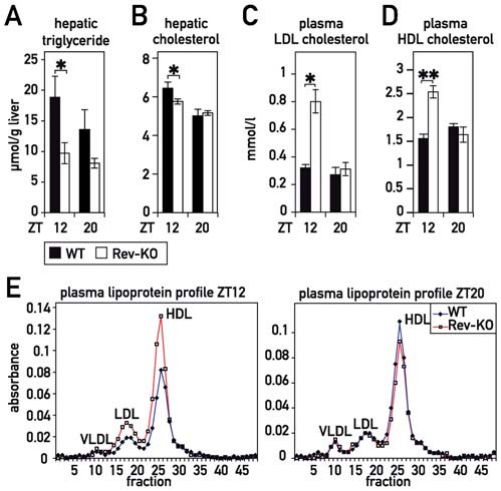
Accumulation of cholesterol and triglycerides in liver and plasma of WT and Rev-KO mice. (A) Total hepatic triglycerides and (B) cholesterol in WT mice (ZT12 *n* = 8, ZT20 *n* = 6) and Rev-KO mice (ZT12 *n* = 10, ZT20 *n* = 4). (C) Plasma LDL-cholesterol and (D) HDL-cholesterol in WT and Rev-KO (*n* = 6 per each data point). (E) Plasma samples from animals humanely killed at ZT12 and ZT20 were pooled (*n* = 6) and lipoproteins separated by fast protein liquid chromatography (FPLC). (A–D) data represent the mean±SEM. *p*-Values: *, <0.05; **, <0.005.

We wished to determine whether the temporal misregulation of SREBP1c also translated to corresponding alterations in the physiology of Rev-KO mice. SREBP1c target genes are implicated in the control of triglyceride synthesis in vivo, and Rev-KO mice indeed showed an altered accumulation of hepatic triglycerides (Figure 3A). Moreover, AACS and HMGCR are key enzymes in cholesterol synthesis, and LDLR is responsible for the uptake of low-density lipoprotein (LDL)-cholesterol from the blood. We hence measured the hepatic cholesterol and the plasma LDL-cholesterol in Rev-KO and WT animals. As shown in Figure 3B, Rev-KO animals had only a modest (albeit statistically significant) decrease in hepatic cholesterol at ZT12. By contrast, plasma LDL-cholesterol levels were highly increased in Rev-KO (Figure 3C), possibly because of a defective LDL-cholesterol uptake by the LDL-receptor (Figure 1D). We also noticed that HDL-cholesterol concentrations were augmented in the plasma of Rev-KO animals specifically at ZT12 (Figure 3D and 3E). Conceivably, increased HDL-cholesterol levels in Rev-KO mice may have been caused by down- and up-regulation of the genes encoding endothelial lipase and apolipoprotein A1 (APOA1), respectively (Figure S5A and S5B). APOA1 is the major lipoprotein in HDL particles [42], and endothelial lipase is an enzyme that catabolizes HDL-cholesterol [43]. However, in contrast to what had been reported previously [44], we did not notice changes in plasma-VLDL between both genotypes, and *ApocIII* mRNA expression in liver was not altered in our Rev-KO or TgRev mouse models (Figure S5C).

In summary, the misregulation of key enzymes involved in cholesterol metabolism in the livers of Rev-KO mice correlate well with the cholesterol phenotype of these animals. We nevertheless want to point out that extra-hepatic tissues may also have contributed to the phenotype (see Discussion).

### *Rev-erbα* Knock-Out Mice Display Decreased Bile Acid Accumulation

**Figure 4:**
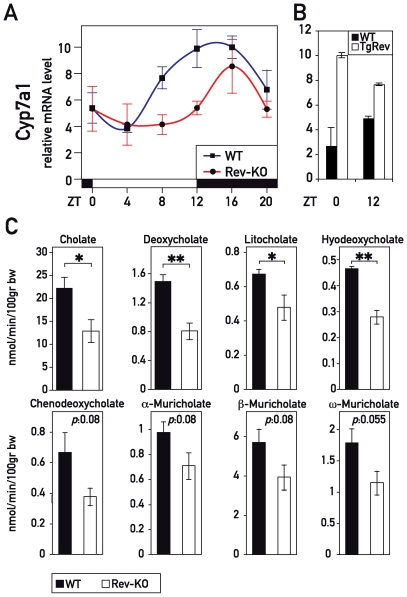
REV-ERBα controls *Cyp7a1* transcription and bile acid accumulation. (A) Temporal *Cyp7a1* mRNA accumulation in the livers of WT, Rev-KO, and (B) TgRev mice. The data represent the mean±SEM (*n* = 4–6). (C) Primary and secondary bile acid levels in gallbladders from WT and Rev-KO mice. Data represent the mean±SEM (*n* = 6). *p*-Values: *, <0.05; **, <0.005.

*Cyp7a1* expression has long been known to be circadian [15], and feeding–fasting rhythms cannot fully account for the daily oscillations in bile acid synthesis [45]. Thus, the circadian clock probably contributes to cyclic *Cyp7a1* expression directly, thereby enabling the anticipation of the need for bile acids. As shown in Figure 4A, both phase and magnitude of *Cyp7a1* expression were severely altered in Rev-KO mice. Moreover, *Cyp7a1* mRNA was constitutively high in TgRev mice (Figure 4B). In Rev-KO animals, the amount of *Cyp7a1* mRNA, integrated over a day, only represented around 60% of the level seen in the WT animals. To investigate whether the 40% decrease has a consequence for bile acid metabolism, we determined the bile acid content in the gallbladder of Rev-KO animals and found a clear decrease in the most abundant primary and secondary bile acids cholate and deoxycholate (Figure 4C). Furthermore, we also noted a general tendency for other bile acid species to accumulate to lower levels in the gallbladders of Rev-KO as compared to control animals (Figure 4C).

### REV-ERBα Represses *Cyp7a1* Transcription through an Indirect Mechanism

**Figure 5:**
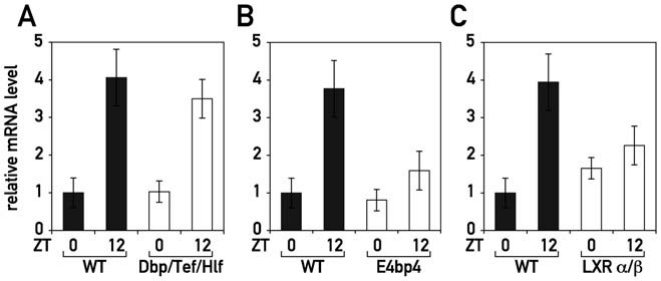
LXR participates in the circadian transcription of *Cyp7a1*. Temporal *Cyp7a1* mRNA accumulation was measured in the livers of (A) *Dbp/Tef/Hlf* triple knock-out mice (B) *E4bp4/Nfil3* knock-out mice and (C) *LXR*α/β double knock-out animals. Data represent the mean±SEM (*n* = 4).

Although it has been known for more than 30 years that *Cyp7a1* is expressed in a circadian fashion, the transcription factors responsible for this regulation are still subject to controversy. Our observation that diurnal *Cyp7a1* was dampened in Rev-KO mice and constitutively high in TgRev mice strongly indicated that *Cyp7a1* expression was linked to the circadian oscillator by REV-ERBα. Since REV-ERBα operates as a transcriptional repressor, the underlying mechanism was most likely indirect, and could have been caused by the down-regulation of a repressor that acts on the *Cyp7a1* gene. Our transcriptome profiling studies revealed the basic leucine zipper protein E4BP4/NFIL3 as a plausible candidate for such a repressor (Table S1). As expected for a direct REV-ERBα target gene, *E4bp4/Nfil3* mRNA accumulation oscillated with the expected phase in WT mice (Figure S6A), and was constitutively high and low in Rev-KO mice and TgRev mice, respectively (Figure S6A and S6B). Temporal E4BP4/NFIL3 protein accumulation was found to be altered accordingly in WT and Rev-KO mice (Figure S6C). Since E4BP4/NFIL3 has been shown to repress transcription through the D-element consensus sequence 5′-RTTAGTAAY-3′ (Figure S6E) [46], a sequence also bound by the three PAR bZip transcription factors DBP, HLF, and TEF, it was tempting to speculate that the E4BP4/NFIL3 repressor and PAR bZip activators could have antagonistic effects on *Cyp7a1* transcription. In cotransfection experiments, DBP [47]–[49] and E4BP4/NFIL3 (Figure S6D) indeed activated and repressed, respectively, transcription from the *Cyp7a1* promoter in a dose- and D-element-dependent manner. However, the implication of PAR bZip proteins or E4BP4 in circadian *Cyp7a1* regulation did not withstand scrutiny by loss-of-function experiments. As shown in Figure 5A, *Dbp/Tef/Hlf* triple knock-out (TKO) mice still exhibited circadian *Cyp7a1* accumulation levels similar to those of WT animals (Figure 5A). If E4BP4/NFIL3 directly repressed *Cyp7a1* transcription, *Cyp7a1* mRNA should accumulate to higher levels in *E4bp4/Nfil3*-KO mice at time ZT0, when *Cyp7a1* expression is low and E4BP4/NFIL3 protein highly abundant (Figures 4A and S6C). However, we found that *Cyp7a1* mRNA accumulated to similar levels at ZT0 and was dampened at ZT12 in *E4bp4/Nfil3*-deficient animals (Figure 5B). These in vivo data argue against a putative role of E4BP4/NFIL3 as a direct *Cyp7a1* repressor. An indirect role of E4BP4/NFIL3 in regulating *Cyp7a1* appears to exist nevertheless, as judged by the down-regulation in the *E4bp4/Nfil3*-KO at ZT12 (a time point when E4BP4/NFIL3 is barely expressed; see Figure S6C).

It is known that in rodents changes in CYP7A1 activity parallel those of HMGCR activity, the enzyme that catalyzes the rate-limiting step in de novo cholesterol synthesis [50]. The specific inhibition of HMGCR by compounds such as lovastatin results in decreased *Cyp7a1* mRNA accumulation [51],[52]. This decrease is prevented if mice receive the HMGCR inhibitor together with an infusion of mevalonate, the product of the reaction catalysed by HMGCR [51],[52]. In addition, inhibition of the last step in cholesterol synthesis by the compound AY9944 also decreases *Cyp7a1* expression [53]. Thus circadian endogenous cholesterol synthesis [17],[54] seems to be linked to the regulation of the *Cyp7a1* transcription.

In Rev-KO and TgRev mice the down- and up-regulation of *Hmgcr* expression observed at ZT12 and ZT0, respectively (Figure 1D), can be expected to engender corresponding changes in oxysterol production [55]. This, in turn, might have an impact on *Cyp7a1* transcription by LXR, which is known to be regulated by oxysterol availability [56]. It is thus conceivable, if not likely, that this pathway can impart on circadian *Cyp7a1* transcription via the activation of LXR receptors in spite of unchanged *Lxr*α and *Lxr*β mRNA levels in Rev-KO mice (Figure 4SC). To test if LXRs could be at least in part responsible for circadian *Cyp7a1* transcription, we measured *Cyp7a1* transcript levels in *Lxr*α/*Lxr*β double knock-out mice (LXRα/β) at ZT0 and ZT12. As shown in Figure 5C, control mice showed a 4-fold amplitude in *Cyp7a1* mRNA levels between ZT0 and ZT12, which was absent in LXRα/β-KO mice. This effect was not due to changes in *Rev-erb*α levels (unpublished data). Thus, a lack of LXR activation by oxysterols at ZT12 seemed to be the most likely scenario to explain the dampened *Cyp7a1* mRNA accumulation seen in Rev-KO mice. Similarly, the up-regulation of *Hmgcr* seen at ZT0 in TgRev animals could have led to increased levels of oxysterols and thus an increase of *Cyp7a1* transcription by LXR receptors.

## Discussion

The genome-wide transcriptome analysis of livers from mice carrying *Rev-erb*α loss- and gain-of-function alleles has identified REV-ERBα as a circadian regulator of cholesterol/lipid and bile acid homeostasis.

Cholesterol is an essential molecule for membrane fluidity, and the synthesis of hormones and bile acids. Likewise, bile acids are required to emulsify absorbed lipids into micelles that are taken up by epithelial cells of the intestine and transported to the liver and other target organs. However, excessive cholesterol can lead to pathologies such as gallstone formation and atherosclerosis. At elevated concentrations bile acids are cytotoxic, and they can also function as proliferation signals [57]. To maintain the bile acid/cholesterol equilibrium, the gene encoding the rate-limiting enzyme of bile acid synthesis from cholesterol, *Cyp7a1*, is tightly regulated. Indeed, a myriad of transcription factors has been shown to act on its promoter (for review see [25]). While the regulatory processes are based on substrate and end-product sensing, *Cyp7a1* expression is also known to be subject to circadian control [15].

Our data suggest that the circadian clock component REV-ERBα participates in this process. Indeed, we found that REV-ERBα controls the timing of cyclic accumulation of SREBP in the nucleus, which in turn regulates the temporal expression of HMGCR. In Rev-KO mice, *Hmgcr* misregulation could engender a diminution of oxysterol production and compromise the LXR-oxysterol-mediated transcription of *Cyp7a1* [56]. Our hypothesis is supported by the fact that drugs which inhibit HMGCR activity are known to decrease *Cyp7a1* transcription, and that circadian *Cyp7a1* expression is severely blunted in Lxrα/β knock-out mice (Figure 5C). It will be interesting to directly determine hepatic oxysterol levels in order to support our conjecture that LXR isoforms drive circadian *Cyp7a1* transcription. Unfortunately, as pointed out by Schroepfer [58], the methods necessary for measuring oxysterols in a reproducible fashion are still challenging. In a recent study, Duez and coworkers [32] have postulated that REV-ERBα regulates *Cyp7a1* transcription by repressing the expression of both E4BP4/NFIL3 and small heterodimer partner (SHP). In our study, however, we did not observe a strong misregulation of *Shp* mRNA accumulation in Rev-KO mice and actually found higher levels in TgRev mice at both time points tested (Figure S7). Several previous studies argue against a major role for SHP in *Cyp7a1* regulation as well. First, *Cyp7a1* expression is known to be only mildly up-regulated in livers of *Shp* knock-out mice [59]. Moreover, the feedback repression of *Cyp7a1* by FXR agonists has been reported to persist in a mouse model carrying a hepatocyte-specific disruption of *Fxr* alleles, despite the lack of *Shp* induction [60]. Furthermore, the circadian phase of *Shp* expression does not correlate with that of other REV-ERBα target genes. *Shp* mRNA accumulation peaks at ZT8 in WT mice; REV-ERBα target genes are at their trough at this time-point. Hence, we do not consider REV-ERBα-controlled SHP expression a very likely mechanism accounting for circadian *Cyp7a1* expression.

Our genetic loss-of-function experiments did not support the role of E4BP4/NFIL3 in circadian *Cyp7a1* expression suggested previously by Duez et al. [32]. Although we observed a similar repressive activity of E4BP4/NFIL3 on the *Cyp7a1* promoter in co-transfection experiments (Figure S6D; see also [32]), there was no up-regulation of *Cyp7a1* expression in the livers of *E4bp4/Nfil3* knock-out mice. Hence, we believe that E4BP4/NFIL3 only down-regulates *Cyp7a1* transcription when overexpressed at unphysiologically high levels. Gain-of-function studies, which are notoriously difficult to interpret, have also led to the suggestion that DBP serves as a circadian activator of *Cyp7a1* transcription [47]–[49]. Again, this result did not bear up against loss-of-function data in the corresponding knock-out mice (Figure 5A). We thus believe that neither E4BP4/NFIL3 nor DBP (or its paralogs TEF and HLF) play significant roles as direct regulators of circadian *Cyp7a1* expression. Our loss-of-function data, rather, implicate the nuclear receptors LXRα/β in the circadian regulation of *Cyp7a1* transcription, but we are aware that the biochemical dissection of this network will require additional experiments.

The liver-specificity of the *Rev-erb*α transgene in the TgRev mice made it unlikely that the misregulation of *Cyp7a1* in the liver originated from a dysfunction outside of this organ. We nevertheless decided to analyze the expression of *Fgf15* in Rev-KO mice, as it is known that this hormone is intestinally produced to negatively feed back on *Cyp7a1* transcription [29]. In addition a recent publication showed that in humans, circulating FGF19 (the homologue of FGF15) has a diurnal variation [61]. We found *Fgf15* to be expressed at similar levels in the intestines of WT and Rev-KO mice (Figure S8). Despite an induction of expression at ZT12, *Fgf15* mRNA levels did not display robust circadian oscillations.

The circadian utilization of cholesterol for bile acid synthesis must be counterbalanced by cholesterol synthesis and uptake in order to maintain stable cholesterol levels. In principle, the sterol-dependent processing and activation of the cholesterol-sensing transcription factor SREBP might be expected to suffice for this purpose. However, our study indicates that SREBP processing is also under circadian control exerted by REV-ERBα. Thus, REV-ERBα governs the rhythmic abundance of INSIG2, which in turn influences the diurnal translocation of SREBP to the nucleus where it transactivates its target genes. Interestingly, FXR has been previously shown to transactivate *Insig2a* expression at the beginning of the light phase [62] and therefore it would be interesting to test the contribution of this nuclear receptor in the circadian regulation of *Insig2a* transcription. Thus, FXR and REV-ERBα, by activating and repressing *Insig2a* transcription, respectively, could act antagonistically on the SREBP pathway.

We found that nuclear SREBP accumulation and target gene expression were misregulated in Rev-KO mice despite indistinguishable feeding rhythms, fasting responses, and insulin levels. This finding strongly suggests that REV-ERBα and the circadian clock impact the SREBP pathway by a more direct mechanism. At ZT12, when animals start to feed, REV-ERBα represses *Insig2a*, and SREBP strongly accumulates in the nucleus. Subsequently *Hmgcr* transcription is induced, and cholesterol synthesis will peak shortly thereafter, together with *Cyp7a1* transcription. Through this elaborate control of timing, the bile acid pool will be replenished in coordination with food uptake. Bile acids will thus be sufficiently available to emulsify dietary lipids.

In addition, our transcriptome analysis of Rev-KO mice points to a broader role for this nuclear receptor as a circadian regulator of lipid metabolism. REV-ERBα regulates genes such as *Elovl3*, *Elovl5*, *Lipoprotein lipase*, *Fatty acid binding protein 5*, *Acyl-CoA thioesterase 3*, or *Phosphatidylcholine transfer protein* (Figure S9). Moreover, we have recently shown that REV-ERBα participates in the transcription of the liver-specific miRNA miR-122 [63]. This miRNA is known to function in lipid and cholesterol metabolism [64],[65].

Finally, our in vivo studies do not support the view that REV-ERBα plays a key role in glucose homeostasis, a hypothesis recently proposed by work with HepG2 cells [66]. Although we did notice a mild hyperglycemia at ZT12 in Rev-KO mice (Figure S10E), the circadian expression of *Phosphoenol pyruvate carboxykinase* (*Pepck*) was nearly unaltered, and that of *Glucose 6-phosphatase* (*G6pase*) was only moderately affected (Figure S10A and S10B). Moreover, these genes were not significantly repressed by overexpressed REV-ERBα (B). In addition, the expression of *Glucokinase* (*Gk*) was not affected by *Rev-erb*α loss- and gain-of-function (Figure S10C). Most importantly, no notable differences in blood glucose clearance were observed between WT and Rev-KO mice upon intraperitoneal glucose injection (Figure S10D). Possible explanations for this discrepancy may lie in species-specific functions of REV-ERBα and/or the difficulty to extrapolate from in vitro or tissue culture studies to physiology in the animal.

**Figure 6:**
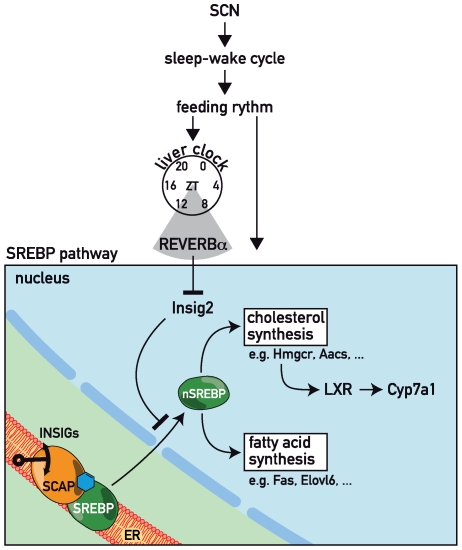
The role of REV-ERBα in circadian lipid and bile acid homeostasis. The cartoon illustrates how REV-ERBα may modulate circadian lipid, cholesterol, and bile acid synthesis by controlling the accumulation of SREBP in the nucleus. In liver hepatocytes, REV-ERBα accumulates to maximal levels at ZT8–ZT12 and represses *Insig2* transcription. Thereby, it promotes the proteolytic activation and nuclear accumulation of SREBP proteins. In turn, the circadian activation of SREBP transcription factors drives the cyclic transcription of *Hmgcr*, encoding the rate-limiting enzyme of cholesterol biosynthesis. As a consequence the levels of oxysterols, which serve as ligands for LXR, also oscillate during the day, and cyclically activated LXR then controls rhythmic *Cyp7a1* transcription.

In conclusion, the data presented in this study suggest that the circadian orphan nuclear receptor REV-ERBα modulates lipid and bile acid metabolism by affecting the SREBP pathway and the regulation of *Cyp7a1* transcription (Figure 6). Our studies, together with work on the substrate-sensing nuclear receptors [67],[68], exemplify how the circadian clock can cooperate with inducible regulatory mechanisms in the daily tuning of lipid and bile acid homeostasis.

## Materials and Methods

### Animal Care and Treatment

All animal studies were conducted in accordance with the regulations of the veterinary office of the State of Geneva and Dutch and Austrian regulations according to criteria outlined in the Guide for the Care and Use of Laboratory Animals prepared by the National Academy of Sciences, as published by the National Institutes of Health. Rev-KO mice have been previously described [8]. Transgenic mice that overexpress hepatic REV-ERBα contain two transgenes [11]. Briefly, the first transgene encodes an HA-epitope-tagged REV-ERBα cDNA controlled by a tetracycline responsive element and the second one expresses a hepatocyte-specific tetracycline-dependent transactivator. In the presence of doxycycline (Dox), transactivator binding is inhibited and thus the *Rev-erb*α transgene remains silent. Dox-fed mice or mice containing only one of the two transgenes were used as control animals. *Lxr*α/*Lxr*β double knock-out and *Dbp/Tef/Hlf* triple knock-out have been previously described [69],[70]. The *E4bp4/Nfil3* knock-out mice have been generated by the laboratory of Andrew Thomas Look (Department of Pediatric Oncology, Dana-Farber Cancer Institute, Harvard Medical School, Boston, Massachusetts). Briefly, exon 2 of the *E4bp4/Nfil3* gene that contains the full coding sequence has been replaced by a neomycin cassette (A.T. Look, personal communication). As expected, no *E4bp4/Nfil3* mRNA could be detected in these mice, see Figure S11. Except for Figure 2B and 2C where mice were fasted for 24 h or were fed a high carbohydrate–no fat diet (Harlan Teklad TD03314), animals received a normal chow diet. Mice were maintained under standard animal housing conditions (12-h light/12-h dark cycle), with free access to food and water.

### Affymetrix Oligonucleotide Microarray Hybridization

Whole-cell liver RNAs from 18 WT and 18 Rev-KO animals (3–4 mo old) humanely killed at ZT12 were used. Three RNA pools of six mice per pool were assembled by mixing equal amounts of RNA. For liver-specific transgenic *Rev-erb*α mice, six animals each for Dox-treated and untreated mice were humanely killed at ZT12 and whole-cell liver RNA was extracted from each animal. Two RNA pools of three animals were assembled by mixing equal amounts of RNA. For Rev-KO, TgRev and WT control animals, 5 µg of pooled RNA were employed for the synthesis of biotinylated cRNA, and 8.75 µg of this cRNA were hybridized to Affymetrix Mouse Genome 430 2.0 array (according to the Affymetrix protocol). To identify differentially expressed transcripts, pairwise comparisons were carried out using Affymetrix GCOS 1.2 software. In the Rev-KO versus WT mice experiment, each of the three experimental samples was compared to each of the three reference samples, resulting in nine pairwise comparisons. This approach is based on the Mann-Whitney pairwise comparison test and allows the ranking of results by concordance, as well as the calculation of significance (*p*-value) for each identified change in gene expression [71],[72]. Genes for which the concordance in the pairwise comparisons exceeded the imposed threshold of 77% (seven out of nine comparisons) were considered to be statistically significant. In addition, we only selected transcripts whose accumulation had an average change of at least 1.5-fold. For these selected probe sets microarray data for the transgenic *Rev-erb*α mice was then extracted (Figure 1A) from the study reported in Kornmann et al. [11].

### Quantitative Reverse Transcriptase-PCR Analysis

cDNA was synthesized from 1 µg of liver whole-cell RNA using random hexamers and Superscript II reverse transcriptase (RT) (Invitrogen) following the supplier's instructions. Five percent of this cDNA was PCR amplified (7900HT Sequence Detection Systems, Applied Biosystems) by using the Sybr Green master mix (Applied Biosystems), and raw threshold-cycle (Ct) values were calculated with SDS 2.0 software (Applied Biosystems). Mean values were calculated from triplicate PCR assays for each sample and normalized to those obtained for *Cyclophillin* and *Eef1a1* transcripts, which served as internal controls [73]. For a list of primers used in this study, see Table S3.

### Western Blotting

Nuclear extracts were prepared by the NUN procedure as described previously [47], and Western blotting was performed according to standard protocols using a mouse anti-SREBP1 monoclonal antibody (BD Biosciences 557036).

### Blood Chemistry

Blood samples were collected at *Zeitgeber* times 12 and 20 (ZT12 and ZT20) in lithium heparin containing tubes (BD Microtainer) and centrifuged for 10 min at 4,500 rpm. Plasma supernatants were kept frozen at −20°C. The levels of HDL- and LDL-cholesterol were measured using a Roche-diagnostics Enzymatic kit for the Hitachi 902 robot according to the manufacturer's instruction. For Rev-KO mice and WT animals (humanely killed at ZT12 and 20) plasma samples were pooled (*n* = 6) and lipoproteins separated by fast protein liquid chromatography (FPLC) on a Superose 6B 10/30 column (Amersham Bioscience). The level of insulin was measured using an ELISA kit (Mercodia 10-1150-01) according to the manufacturer's instruction, from blood samples collected at ZT4, ZT12, and ZT16.

### Bile Analysis

Mice were anesthetized by intraperitoneal injection of Hypnorm (fentanyl/fluanisone 1 ml/kg) and diazepam (10 mg/kg). Bile was collected during 30 min by cannulation of the gallbladder, and total bile acids were measured enzymatically [74]. Total gallbladder cholesterol was measured according to Kruth et al [75]. For the determination of specific bile acid species, bile acids extracted from bile [76] were analyzed by gas chromatography mass spectrometry (GC-MS).

### Hepatic Cholesterol and Triglycerides

Livers were homogenized and lipids extracted as described by Bligh and Dyer [76]. Hepatic triglyceride and cholesterol concentrations were measured using an enzymatic kit (Roche Molecular Biochemicals, Mannheim, Germany) according to the manufacturer's instruction.

### Accession Numbers

The GenBank (http://www.ncbi.nlm.nih.gov/Genbank) accession numbers for the genes and gene products discussed in this paper are *Aacs* (NM_030210); *Acot3* (NM_134246); *Apoa1* (NM_009692); *ApoCIII* (NM_023114); *Bmal1* (NM_007489); *Cyp7a1* (NM_007824); *Dbp* (NM_016974); *E4bp4/Nfil3* (NM_017373); *Elovl3* (NM_007703); *Elovl5* (NM_134255); *Elovl6* (NM_130450); *Fabp5* (NM_010634); *Fads2* (NM_019699); *Fas* (NM_007988); *Fgf15* (NM_008003); *G6pc* (NM_008061); *Glucokinase* (NM_010292); *Hlf* (NM_172563); *Hmgcr* (NM_008255); *Insig1* (NM_153526); *Insig2* (NM_133748); *Ldlr* (NM_010700); *Lipase endothelial* (NM_010720); *Lpl* (NM_008509); *Lxrα* (NM_013839); *Lxr*β (NM_009473); *Pctp* (NM_008796); *Pepck* (NM_011044); *Rev-erb*α (NM_145434); *Scap* (NM_001001144); *Shp* (NM_011850); *Srebp1* (NM_011480); *Srebp2* (NM_033218); *Tef* (NM_017376).

The ArrayExpress repository (http://www.ebi.ac.uk/arrayexpress) accession number for the microarray data is E-TABM-726.

## Supporting Information

Figure S1 **Temporal expression of** ***Rev-erb*** **α and** ***Bmal1*** **mRNA in liver of WT and Rev-KO mice, as monitored by quantitative RT-PCR.** mRNA levels were determined around the clock from pooled whole-cell RNAs (four to six animals per time point).
(0.45 MB EPS)

Figure S2 **Possible mechanisms accounting for the four major expression patterns identified in** **Figure 1A** **.** In the left diagrams, target gene expression (*y*-axis) is schematically plotted against REV-ERBα concentration. The diagrams to the right depict possible protein-DNA and protein-protein interactions that might account for the observed expression patterns. Class I: The genes belonging to this class are likely to be direct REV-ERBα targets. Their expression is upregulated in the Rev-KO mice, and repressed equally or more efficiently in TgRev mice as compared to WT mice. Class II: These genes may also be direct REV-ERBα targets, but their transcription is less efficiently repressed in an excess of REV-ERBα. Conceivably, unsuccessful competition of promoter-bound REV-ERBα with free REV-ERBα (or REV-ERBα bound nonspecifically to other DNA sequences) for corepressor complexes may account for the derepression at high REV-ERBα concentrations. The competition of promoter-bound and unbound transcriptional regulators for rate-limiting cofactors was termed squelching by Gill and Ptashne [77]. Class III: The transcription of these genes is indirectly regulated by REV-ERBα, because of the down regulation of a repressor (i.e., E4BP4/NFIL3). Class IV: Indirect REV-ERBα target genes (see class III) whose repressor is encoded by a class II gene (i.e., squelched at high REV-ERBα concentrations). Obviously, many other, more complex mechanisms may also account for the expression patterns of class II and class IV transcripts.
(0.74 MB EPS)

Figure S3 **Analysis of circadian mRNA accumulation profiles of the genes found to be up- and down-regulated in Rev-KO mice.** Temporal accumulation patterns of the 76 and 60 transcripts listed in Table S1 and S2 (represented by 97 and 81 probe sets, respectively) that were found to be ≥1.5-fold up- and down-regulated in Rev-KO mice at ZT12. These profiles were retrieved from the Gene Expression Omnibus GSE11923 and http://bioinf.itmat.upenn.edu/circa database [10]. This database contains the circadian analysis of the hepatic transcriptome at a 1-h resolution. (A) Phase distribution of the 454 probe sets found to be circadian in WT mice. (B) Phase distribution of the 56 and (C) 35 probe sets found to be up- or down-regulated in Rev-KO mice and circadian in WT animals (with an amplitude of ≥2, see Kornmann et al. for details [11]). The up- and down-regulated probe sets in Rev-KO mice are enriched 55- and 43-fold, respectively, in circadian probe sets (*p*<10^−10^).
(0.40 MB EPS)

Figure S4 **Temporal hepatic expression of transcripts specified by (A)** ***Srebp2*** **, (B)** ***Scap*** **, and (C)** ***Lxr*** **α and** ***Lxr*** **β in WT and Rev-KO mice, as monitored by quantitative RT-PCR.** mRNA levels were determined around the clock from pooled whole-cell RNAs (four to six animals per time point).
(0.53 MB EPS)

Figure S5 **Temporal hepatic expression of transcripts specified by (A)** ***Lipase endothelial*** **, (B)** ***Apoa1*** **, (C)** ***Apoc3*** **in WT, Rev-KO, and TgRev mice.** In the left panels mRNA levels were quantified as described in the legend to Figure S4. The right histograms show mean values±standard error of the mean (SEM) obtained from individual animals at ZT0 and ZT12 (*n* = 3 for WT mice, and *n* = 4 for TgRev mice).
(0.43 MB EPS)

Figure S6 **REV-ERBα controls the circadian expression of the E4bp4/Nfil3 repressor.** Temporal *E4bp4/Nfil3* mRNA accumulation in the livers of (A) Rev-KO and (B) TgRev mice. (C) Immunoblot analysis of E4BP4/NFIL3 accumulation at ZT0, ZT4, ZT8, ZT12, ZT16, and ZT20 in livers of WT and Rev-KO mice. The same blot was probed with an antibody against the constitutively expressed splicing factor U2AF^65^ (loading control). (D) Cotransfection of an E4BP4/NFIL3 expression vector with *Cyp7a1*-luciferase reporter genes harboring an intact D-element (black columns) or two mutated D-elements (mut1, white columns; mut2, grey columns). The sequences of intact D-elements and mutated D-elements are depicted in (E).
(1.75 MB EPS)

Figure S7 **Temporal hepatic expression of transcripts specified by the SHP in WT, Rev-KO, and TgRev mice.** In the left panels mRNA levels were quantified as described in the legend to Figure S4. The right histogram shows mean values±SEM obtained for individual animals at ZT0 and ZT12 (*n* = 3 for WT mice, and *n* = 4 for TgRev mice).
(0.47 MB EPS)

Figure S8 **Temporal intestinal (Ileum) expression of transcripts specified by the fibroblast growth factor 15 in WT and Rev-KO, as monitored by quantitative RT-PCR. mRNA levels were determined around the clock.** The data are represented as mean values±SEM (*n* = 4 animals per time point).
(0.31 MB EPS)

Figure S9 **Expression of genes involved in hepatic lipid metabolism and transport in WT and Rev-KO mice.** The temporal hepatic expression of transcripts was determined by Northern blot hybridization (*Elovl3* and *Elovl5*), or quantitative RT-PCR (*Lpl*, *Fabp5*, *Pctp*, *Acot3*) from pooled whole-cell RNAs from four to six animals per time point.
(2.83 MB EPS)

Figure S10 **REV-ERBα plays a minor role in glucose homeostasis.** Temporal expression of (A) *Pepck*, (B) *G6pc*, and (C) *Gk* mRNA in liver of WT, Rev-KO, and TgRev mice. (A–C), left panels, mRNA levels were determined around the clock from pooled whole-cell RNAs (four to six animals per time point) or (A–C), right panel, from individual animals at ZT0 and ZT12 (data represent mean values±SEM; WT, *n* = 3; TgRev, *n* = 4). (D) Glucose tolerance test on WT and Rev-KO mice (data represent mean values±SEM, *n* = 9). (E) Blood glucose concentration determined at ZT12 and ZT20 for WT and Rev-KO mice (data represent means±SEM; ZT12, WT, *n* = 14 and Rev-KO, *n* = 12; ZT20, WT, *n* = 8 and Rev-KO, *n* = 8).
(0.45 MB EPS)

Figure S11 **Temporal hepatic expression of** ***E4bp4/Nfil3*** **transcripts in WT and E4bp4-KO mice, as monitored by quantitative RT-PCR.** Data represent the mean±SEM (*n* = 4), number values below detection level.
(0.29 MB EPS)

Table S1 **Genes up-regulated in Rev-KO mice.**
(0.03 MB XLS)

Table S2 **Genes down-regulated in Rev-KO mice.** In Table S1 and S2 the fold changes (FC) are the average ratios between transcript accumulation levels (Rev-KO/WT) of all nine comparisons (see experimental procedures for the analysis of Affymetrix microarray data). The estimated fold changes were verified by Northern blot (Nb) or quantitative RT-PCR experiments. The minus signs in Table S2 indicate that these transcripts accumulated to lower levels in Rev-KO mice than in WT mice.
(0.03 MB XLS)

Table S3 **Nucleotide sequences of gene-specific primers used in this study.**
(0.02 MB XLS)

## Abbreviations

AACS: acetoacetyl-CoA synthetase
CYP7A1: cytochrome P450 7α-hydroxylase
DBP: albumin D-site-binding protein
HLF: hepatic leukemia factor
Hmgcr: Hmg-CoA reductase
INSIG: insulin-induced gene
LDL: low-density lipoprotein
Rev-KO: *Rev-erb*α knock-out mice
RT: reverse transcriptase
SCAP: SREBP cleavage activating protein
SCN: suprachiasmatic nucleus
SEM: standard error of the mean
SHP: small heterodimer partner
SREBP: sterol regulatory element-binding protein
TEF: thyrotroph embryonic factor
TgRev: transgenic mice that overexpress hepatic REV-ERBα throughout the day
WT: wild type
ZT: Zeitgeber time
